# Effect of UV Irradiation on Properties and Characteristics of Fish Gelatin-Based Film Containing Linoleic Acid and Ferrous Chloride

**DOI:** 10.3390/polym17182512

**Published:** 2025-09-17

**Authors:** Wipawee Theerawitayaart, Kullaya Poomithorn, Krisana Nilsuwan, Ponsatit Sookchoo, Soottawat Benjakul, Thummanoon Prodpran

**Affiliations:** 1 Food Packaging Technology Program, Faculty of Agro-Industry, Prince of Songkla University, Hat Yai, Songkhla 90110, Thailand; wipawee.mdd@gmail.com (W.T.); pornsatit.s@psu.ac.th (P.S.); 2 Center of Excellence in Bio-Based Materials and Packaging Innovation, Faculty of Agro-Industry, Prince of Songkla University, Hat Yai, Songkhla 90110, Thailand; kullaya.p@psu.ac.th; 3 International Center of Excellence in Seafood Science and Innovation, Faculty of Agro-Industry, Prince of Songkla University, Hat Yai, Songkhla 90110, Thailand; krisana.n@psu.ac.th (K.N.); soottawat.b@psu.ac.th (S.B.)

**Keywords:** gelatin film, UV-irradiation, linoleic acid, molecular modification, water-vapor barrier

## Abstract

This study investigated the combined effects of linoleic acid (LA) incorporation and UV irradiation in the presence and absence of ferrous chloride (FeCl_2_) on the properties and characteristics of fish skin gelatin films. UV irradiation was implemented at different intensities (10,000–40,000 lux) and with different exposure times (1 and 5 min) by two different methods: irradiating the film-forming solution before casting (S-UV) versus irradiating the pre-cast film (F-UV). The UV treatment significantly increased the elastic modulus (EM) while decreasing the tensile strength (TS), elongation at break (EAB), and water-vapor permeability (WVP) of the films (*p* < 0.05), irrespective of the irradiation method used. This effect became more pronounced with higher UV intensity and longer exposure times. When both LA and FeCl_2_ were present, UV irradiation promoted the formation of non-disulfide covalent bonds, leading to increased cross-linking. This cross-linking improved the film’s strength and decreased its WVP, although it did cause the films to become yellowish. Fourier-transform infrared spectroscopy (FTIR) confirmed interactions between the gelatin and LA, indicated by a decrease in the intensity of Amide-A, Amide-I, and Amide-II bands. A key finding suggested that UV irradiation, combined with LA/FeCl_2_ incorporation, could significantly enhance the properties of fish skin gelatin films, especially their water-vapor barrier. The study’s novelty lies in demonstrating that applying the UV treatment to either the film solution or the final film yields similar results, providing flexibility in the manufacturing process.

## 1. Introduction

Polymers are widely used for food packaging, but growing concerns about pollution are driving a shift toward more sustainable options. Biodegradable films derived from biopolymers are emerging as a viable alternative to traditional petroleum-based materials. These films are typically made from proteins, lipids, and polysaccharides [1,2]. Protein-based films have gained significant attention for their potential as eco-friendly packaging due to their excellent film-forming capabilities. Among these, films made from fish skin gelatin are particularly promising. They are appealing for food packaging applications because of their notable transparency and ability to be combined with beneficial bioactive compounds [3,4]. Despite their advantages, gelatin films have a major drawback due to their inherent hydrophilic nature. This high affinity for water causes them to swell, degrade, or dissolve when exposed to moisture. This sensitivity to humidity negatively impacts their performance, leading to dimensional instability, lower mechanical strength, and reduced barrier properties, which ultimately decreases the shelf life of packaged products. These weaknesses limit their use in food packaging, where robust barrier properties are essential for preserving product quality and extending shelf life [1,4,5,6].

To lower the hydrophilic nature of gelatin films, incorporating hydrophobic substances like fatty acids, oils, and waxes is a common method for reducing the water-attracting properties of gelatin films [5,6]. Studies have shown that adding different fatty acids, including palmitic, stearic, and linoleic acids, to biopolymer films (especially gelatin-based ones) improves their hydrophobicity, water-vapor barrier, and flexibility [7,8]. For example, a study by Nor Amalini et al. [8] found that the addition of sucrose esters of palmitic and stearic acid to gelatin films significantly lowered their water-vapor permeability and solubility, while also increasing their opacity.

Additionally, the use of oxidized lipids, specifically the reactive aldehydes and ketones they produce, has been shown to chemically alter gelatin chains, which makes the gelatin more water-repellent [9,10]. This modification also enhances the resulting gelatin film’s mechanical strength and its ability to act as a water-vapor barrier [11,12]. A more recent study by Theerawitayaart et al. [11] revealed that films made from gelatin that was chemically modified with oxidized linoleic acid (OLA) were more flexible and had a better water-vapor barrier than films where OLA was just directly added at low concentrations. The modified films also had better long-term stability, particularly in resisting yellow discoloration and rancidity [12].

Ultraviolet (UV) irradiation, a versatile, non-thermal method, has been used as a method for protein modification. This process enhances the barrier and mechanical characteristics of packaging films, particularly those made from gelatin and other biodegradable sources. The final effects of this modification are highly dependent on both the specific proteins and the methods used to create the films [13,14,15,16,17]. De Vargas et al. [18] demonstrated that UV irradiation is a powerful treatment for inducing the cross-linking of gelatin chains, providing films with increased tensile strength and Young’s moduli but decreased elongation and water solubility. This irradiation method has been successfully used to enhance the functional properties of protein films by cross-linking mechanisms [18]. Particularly, the electromagnetic radiation of UV is absorbed by double bonds and aromatic rings, causing free radical formation in amino acids. This can lead to the formation of intermolecular covalent bonds [14,15,16,17].

While previous studies have explored the use of UV irradiation for protein modification, and others have investigated the incorporation of lipids and oxidized lipids into gelatin films, there is a lack of information on the combined effect of these treatments. Specifically, the influence of modifying fish gelatin with fatty acids in the presence of a pro-oxidant like ferrous chloride (FeCl_2_) in combination with UV irradiation has not been thoroughly reported. The UV treatment of gelatin incorporated with fatty acids, especially in the presence of metal as a pro-oxidant, might enhance the oxidation of fatty acids. As a consequence, oxidized lipids induced by UV irradiation at an appropriate level might, in situ, modify gelatin via covalent attachment to gelatin molecules, thus affecting the properties of gelatin films, not only their water-barrier property but also other properties.

Therefore, the purpose of this study was to represent an innovative approach by investigating the synergistic effects of UV irradiation and the incorporation of linoleic acid (LA) with ferrous chloride (FeCl_2_) as a pro-oxidant to enhance the functional properties of a biodegradable fish gelatin film. Particularly, the research aimed to evaluate the effects of the UV irradiation at different intensities and exposure times, by using two different methods, including applying UV to the film-forming solution before casting (S-UV) and to the pre-cast film (F-UV), on the properties and characteristics of final fish gelatin films containing LA without and with FeCl_2_ addition. The film samples were analyzed for their physico-chemical properties, such as mechanical properties, water-vapor permeability, and optical properties, and some characteristics including molecular interactions via FTIR analysis, morphology, thermal properties, and protein pattern.

Overall, our findings demonstrate that the combination of UV irradiation with LA and FeCl_2_ significantly improves the film’s water-vapor barrier and mechanical properties, primarily due to enhanced cross-linking. The stage of UV treatment (before or after casting) had no significant impact on the film’s properties. In conclusion, this research provides a promising and effective method for creating enhanced-performance, bio-based packaging materials.

## 2. Materials and Methods

### 2.1. Fish Gelatin and Chemicals

Gelatin powder from tilapia skin, having a bloom strength of 240, was purchased from Lapi Gelatine S.p.A (Empoli, Italy). Ferrous chloride (FeCl_2_), linoleic acid, glycerol, sodium dodecylsulfate (SDS), Tris hydrochloride, β-mercaptoethanol (β-ME), and Coomassie blue R-250 were acquired from Sigma-Aldrich (St. Louis, MO, USA). Methanol and acetic acid were obtained from Merck (Darmstadt, Germany). All chemicals were of Analytical Reagent (AR) grade, with the purity specifications for laboratory testing procedures met.

### 2.2. Preparation of Film-Forming Solutions

A film-forming solution (FFS) was prepared following the procedure reported by Tongnuanchan et al. [19]. Firstly, gelatin of a designated amount was mixed with distilled water to obtained a protein concentration of 3% (*w*/*v*), and stirred continuously in a controlled-temperature water bath at 60 °C for 30 min, followed by cooling down to room temperature. Glycerol (30% (*w*/*w*) based on protein content) was then added into the gelatin solution at room temperature (28–30 °C) and further stirred for 5 min. The solution was supplemented with linoleic acid (LA) at a concentration of 30% (*w*/*w*) based on protein content. The mixture was stirred at room temperature for 30 min and homogenized using a homogenizer (IKA Labortechnik homogenizer, Selangor, Malaysia) for 3 min at 22,000 rpm. Thereafter, the ferrous chloride (FeCl_2_) was added into the FFS at 1.5% (*w*/*w*) based on protein content. After stirring for 30 min, the emulsions were placed in a vacuum pump (Diaphragm vacuum pump, Wertheim Germany) at room temperature for 30 min to remove air bubbles. The control gelatin film was formulated identically, with the exception of leaving out LA and FeCl_2_.

### 2.3. Preparation of Gelatin Film Irradiated with UV

UV irradiation was implemented using two methods: (1) the FFS was irradiated prior to film casting (S-UV); (2) the pre-cast film was UV irradiated (post-film treatment) (F-UV). The UV chamber was equipped with a high-pressure mercury lamp (400 W) (Model 4/120, Brilliant Token Co., Ltd., Bangkok, Thailand), providing a wavelength of 365 nm. Before irradiation, the lamp was turned on for 15 min to warm up.

For the FFS UV treatment, the solutions were placed in the UV chamber at different distances from the UV lamps of 58, 40, 32, and 27 cm to obtain the intensities of 10,000, 20,000, 30,000, and 40,000 lux, respectively. The UV light’s intensity was measured using a digital lux meter (LX1010B, Hefei Vetus Electronic Technology Co., Ltd., Anhui, China). At each intensity, the FFS was irradiated for 1 and 5 min in air. After being irradiated under the designated condition, a precise amount of FFS (46.3 ± 0.01 g) was introduced to a square, rimmed, silicon resin substrate (17 × 17 cm^2^) for molding. Subsequently, the casted FFS was air-blown at room temperature for 12 h to remove water. The films were placed in an environmental chamber (WTB Binder, Tuttlingen, Germany) to dry for 48 h. The drying conditions were maintained at 25 ± 0.5 °C and 50 ± 5% relative humidity (RH). The resulting films were manually and carefully peeled off from the casting plate. Films obtained by this UV irradiation method were named “S-UV”.

For the pre-cast film UV treatment, the pre-cast films were irradiated by UV using the same intensity and exposure time. UV-treated film samples obtained by this method are referred to as “F-UV”. Control films were prepared identically to the others, but without UV irradiation, for both the FFS and pre-cast films.

### 2.4. Analysis of Film Properties

To ensure consistent results, the film samples were equilibrated at 25 ± 0.5 °C and 50 ± 5% RH for 48 h before analysis.

#### 2.4.1. Thickness of the Film

The film’s thickness was precisely gauged using a digital micrometer (Mitutoyo, Model IDC112PM, Serial No. 00320, Mitutoyo Corp., Kawasaki-shi, Japan). Overall, ten replicates were taken as the average for each film at ten random locations. The average thickness value was recorded.

#### 2.4.2. Tensile Mechanical Properties

The mechanical properties of each film were evaluated using a Universal Testing Machine (Lloyd Instrument, Hampshire, UK). Following the ASTM D882 standard, with slight modifications from the protocol described by Tongnuanchan et al. [19,20], the tensile strength (TS), elongation at break (EAB), and elastic modulus (EM) were assessed. Film samples (2 cm × 5 cm) were secured between grips with an initial 3 cm separation, and a cross-head speed of 30 mm/min was applied. The tests were performed with a 100 N load cell on a minimum of ten samples per treatment.

#### 2.4.3. Water-Vapor Permeability (WVP)

A gravimetric test, adhering to the ASTM E96-80 standard [21], was employed to assess the water-vapor permeability (WVP) of the gelatin films. The experimental setup involved an aluminum cup containing 20 g of dried silica gel (0% RH), which acts as a desiccant. A 4 × 4 cm^2^ gelatin film was mounted over the cup’s opening and sealed securely using a neoprene rubber gasket and silicone grease, fastened with screws. The entire assembly was weighed and then transferred to a controlled chamber (25 °C, 50% RH). Weight gain was measured hourly over a 10 h period. Three samples of each film treatment were tested. The slope from the plot of weight gain over time was used to calculate the WVP. The WVP of the film sample was calculated as follows:

(1) $$WVP\left({\mathrm{gm}}^{-1}s^{-1}{\mathrm{Pa}}^{-1}\right)=\frac{w\cdot l}{At∆P}$$

where *w* is the weight gain of the cup (g), *l* is the thickness of the film (m), *A* is the total test area of the film (m^2^), *t* is the conditioning time (s), and ∆*P* is the vapor pressure difference between the outside and inside of the film (Pa).

#### 2.4.4. Color of the Film

The color of the film is a crucial quality attribute for packaging materials, as it influences consumer acceptance and the esthetic presentation of the final product. The L*, a*, and b* values of the films were determined with a D65 standard illuminant using a Hunter Associates Laboratory, Inc. CIE colorimeter. The colorimeter was calibrated using black and white plates. For each film sample, three readings were taken at different locations, with each reading performed in triplicate. The total color difference, denoted as ∆E*, was subsequently calculated using the following equation [13]:

(2) $$∆E^{*}=\sqrt{{\left(∆L^{*}\right)}^{2}+{\left(∆a^{*}\right)}^{2}+{\left(∆b^{*}\right)}^{2}}$$

where ∆L*, ∆a*, and ∆b* represent the differences in values between each color parameter of the film samples and that of the white standard color plate (L* = 93.45, a* = −0.81, b* = 0.33) used as the film background.

#### 2.4.5. Transparency Value of Film

The transparency value of the film was measured using a UV–vis spectrophotometer (UV-1800, Shimadzu, Kyoto, Japan) as tailored by Shiku et al. [22]. The transparency of a film is a crucial property for food packaging, as it affects the visual appeal and quality perception of the product by the consumer. The film’s transparency was assessed using visible light at a wavelength of 600 nm. The calculation was based on the film’s thickness (*x*) and the fraction of light transmitted at 600 nm (*T*_600_) according to the following equation:

(3) $$\mathrm{Transparency value}=\frac{-\log T_{600}}{x}$$

This equation shows that a larger transparency value corresponds to more opacity (less transparency). The transparency value measurement provides insight into the film’s microstructure, as light scattering can reveal the presence of dispersed components and aggregates, thus complementing the SEM and FTIR analyses.

### 2.5. Characterization of Selected Films

Both S-UV- and F-UV-treated films prepared using the selected UV-irradiation conditions that rendered the lowest WVP were chosen for characterization. For this, UV irradiation at 40,000 lux for 5 min was implemented to prepare the S-UV- and F-UV-treated films with three different conditions including (1) without LA and FeCl_2_, (2) with 30% LA, and (3) with 30% LA and 1.5% FeCl_2_. The obtained UV-treated samples prepared under corresponding conditions 1, 2, and 3 (being coded as S-UV1, S-UV2, S-UV3, F-UV1, F-UV2, and F-UV3) were characterized and compared with those of the control gelatin film without UV treatment and LA and FeCl_2_ addition. Before characterization, the selected film samples were conditioned in a desiccator containing P_2_O_5_ for 2 weeks at room temperature to obtain the most dehydrated film [23].

#### 2.5.1. Scanning Electron Microscopy (SEM)

The film’s microstructure was examined via scanning electron microscopy (SEM), specifically using a Quanta 400 from FEI (Eindhoven, The Netherlands). The instrument’s acceleration voltage was set to 15 kV. Cross-section samples were prepared by cracking them in liquid nitrogen. Both surface and cross-section specimens were secured to a bronze holder with electrically conductive carbon tape. A gold coating was applied with an SPI-Module sputter coater (Structure Probe, Inc., West Chester, PA, USA) to ensure sufficient conductivity for imaging.

#### 2.5.2. Fourier-Transform Infrared (FTIR) Spectroscopic Analysis

The gelatin films were examined via Fourier-transform infrared (FTIR) spectroscopy. A Bruker Equinox 55 FTIR spectrometer (Bruker Co., Ettlingen, Germany) was used to collect spectra over a wavenumber range of 400 to 4000 cm^−1^. The instrument was configured with a spectral resolution of 4 cm^−1^, and each measurement consisted of 32 scans. For sample analysis, a horizontal attenuated total reflection (ATR) crystal cell made of zinc selenide (ZnSe) was employed. This crystal, set at a 45° angle, was 80 mm long, 10 mm wide, and 4 mm thick (PIKE Technology Inc., Madison, WI, USA). All measurements were conducted at a controlled temperature of 25 °C. To ensure data consistency, the collected spectra were normalized against a background spectrum of air. This methodology is based on the work of Nuthong et al. [24].

#### 2.5.3. Thermogravimetric Analysis (TGA)

The degradation temperature and weight loss of selected films upon heating from 50 to 800 °C at a rate of 10 °C/min were determined using a thermogravimetric analyzer (TGA7, PerkinElmer, Norwalk, CT, USA) as detailed by Nuthong et al. [24]. Film samples were analyzed in a nitrogen environment with a flow rate of 20 mL/min.

#### 2.5.4. SDS–Polyacrylamide Gel Electrophoresis (SDS–PAGE)

The pattern of proteins in selected gelatin films was determined using sodium dodecyl sulfate–polyacrylamide gel electrophoresis (SDS–PAGE) as described by Laemmli [25] under reducing conditions. A modified protocol based on Theerawitayaart et al. [10] was used to prepare the samples. Each 200 mg film sample was solubilized by mixing it with 10 mL of 1% (*w*/*v*) SDS and stirring at room temperature for 12 h. A Hettich Centrifuge (MIKRO-20, D-78532, Tuttlingen, Germany) was used to centrifuge the mixtures at 3000× *g* for 5 min, after which the supernatants were collected. These were then mixed with a sample buffer (20% (*v*/*v*) glycerol, 0.5 M Tris-HCl, pH 6.8, with 4% (*w*/*v*) SDS and 10% (*v*/*v*) β-ME) in a 1:1 volume ratio. A 4% stacking gel and a 10% running gel were loaded with 15 µg of protein, which was quantified using the Biuret method [26]. Electrophoresis was performed at a constant current of 15 mA with a Mini Protein II unit (Bio-Rad Laboratories, Inc., Richmond, CA, USA). The gel was subsequently stained with 0.05% (*w*/*v*) Coomassie blue R-250 in a solution of 15% (*v*/*v*) methanol and 5% (*v*/*v*) acetic acid. The staining process was followed by destaining with a mixture of 30% (*v*/*v*) methanol and 10% (*v*/*v*) acetic acid until the gel’s background was clear. The protein molecular weights were estimated using a high-molecular-weight marker.

### 2.6. Statistical Analyses

Experiments were performed in triplicate. For statistical evaluation, SPSS software (version 22 for Windows; SPSS Inc., Chicago, IL, USA) was used. The data were analyzed using a one-way ANOVA, and a Duncan’s multiple-range test was used to determine significant differences between means. A *p*-value of less than 0.05 was considered statistically significant.

## 3. Results and Discussion

### 3.1. Properties of Fish Skin Gelatin Films with Incorporation of Linoleic Acid (LA) and Ferrous Chloride (FeCl_2_) as Affected by UV Irradiation

#### 3.1.1. Thickness and Mechanical Properties

**Figure 1:**
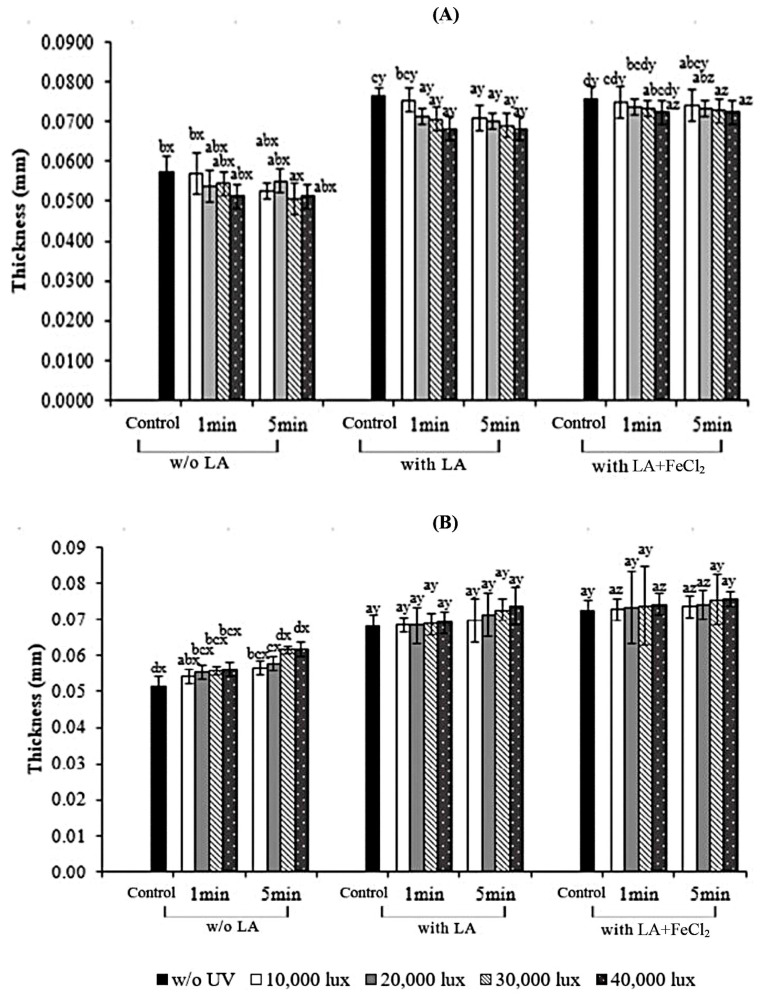
Effect of UV treatment by different irradiation methods on thickness of gelatin films with and without 30% linoleic acid (LA) and 1.5% ferrous chloride (FeCl_2_). (**A**) S-UV: the film-forming solution was irradiated before casting; (**B**) F-UV: the pre-cast films were irradiated. The letters (a–d) and (x, y, z) on the bars indicate significant differences (*p* < 0.05).

The thickness values of all the film samples are shown in Figure 1. Films without LA incorporation had lower thickness than those supplemented with LA and LA + FeCl_2_ (*p* < 0.05), regardless of UV treatment application. LA-supplemented films and LA + FeCl_2_-supplemented films showed higher thickness (*p* < 0.05). The thickness value was slightly increased when LA was incorporated. No marked difference was found when FeCl_2_ was added into films containing LA (*p* ≥ 0.05). The increased thickness might be related to the film-forming composition as associated with the increase in the solid content of the film [27]. The LA used in the formulation mainly decreased the compactness of the polymer matrix, in which interaction and conjunction between protein molecules were impeded as indicated by the higher thickness. Small differences in thickness were observed between irradiated and non-irradiated films. Moreover, a similarity in thickness was observed between the S-UV and F-UV films.

**Figure 2:**
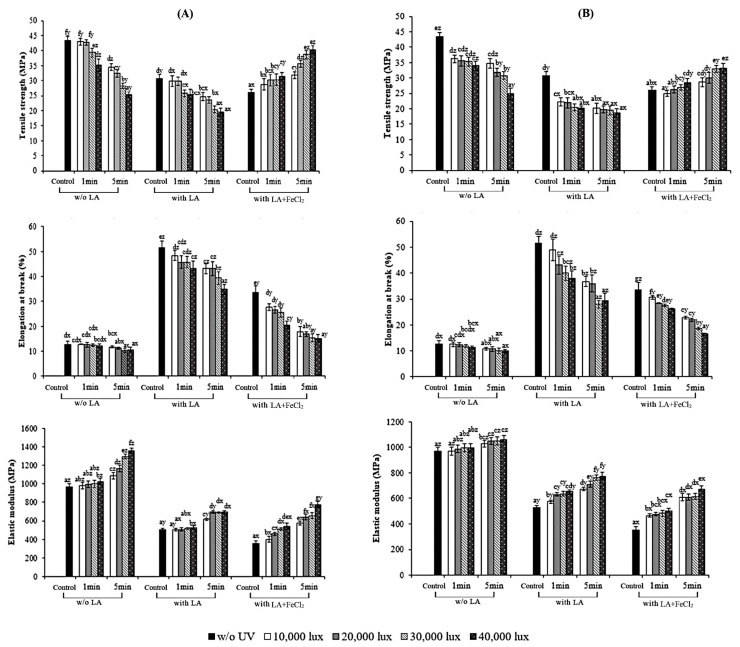
Effect of UV treatment by different irradiation methods on mechanical properties of gelatin films with and without 30% linoleic acid (LA) and 1.5% ferrous chloride (FeCl_2_). (**A**) S-UV: the film-forming solution was irradiated before casting; (**B**) F-UV: the pre-cast films were irradiated. The letters (a–g) and (x, y, z) on the bars indicate significant differences (*p* < 0.05).

The mechanical properties of the control as well as S-UV and F-UV films containing or not containing LA and FeCl_2_ are depicted in Figure 2. The incorporation of LA increased the EAB while reducing the TS and EM of the resulting films compared to the control film (*p* < 0.05). This effect was consistent across all films prepared by different UV treatment methods. The findings indicated that the addition of LA had a plasticizing effect since oil droplets might interfere with the close-knit interactions between gelatin chains, reducing their aggregation and crating more free volume within the film’s network [27,28,29]. The disruption created a more pliable film, which was shown by its higher EAB but lower TS and EM. This observation aligned with previous work by Limpisophon et al. [30] on gelatin films from blue shark skin. Chen et al. [5] also reported that the addition of stearic acid and lauric acid to protein-based films significantly decreased the TS but increased the EAB.

In films containing only LA, it was noted that the TS and EAB continuously decreased as the UV intensity and irradiation time increased (*p* < 0.05). UV irradiation might induce the formation of reactive oxygen species (ROS) and other radicals. These radicals then attack specific parts of the protein, like tyrosine and phenylalanine amino acids, which can cause the protein chains to break down [31]. This might result in the lowered TS and EAB as well as slightly decreased thickness. For films containing LA and FeCl_2_, in the presence of LA (30% (*w*/*w*) of protein), the incorporation of FeCl_2_ increased the TS but decreased the EAB in comparison with that containing only LA (*p* < 0.05). It was suggested that the addition of FeCl_2_ to gelatin film containing LA could induce lipid oxidation and thus the formation of aldehyde moieties from lipid peroxidation such as malondialdehyde, hydroxynonenal, and acrolein. Those compounds could covalently bind to protein chains [32]. Similar results were observed, regardless of UV intensity and irradiation time.

Nevertheless, for films containing both LA and FeCl_2_, the continuous increases in both TS and EM but decrease in EAB were observed when the UV intensity and irradiation time increased (*p* < 0.05). Yamamoto et al. [31] also reported that UV irradiation can induce both the scission and cross-linking of protein chains due to reactive species generated by oxidation reaction. At a high UV intensity, the oxidation of LA in the presence of a catalyst, Fe^2+^, took place and the aldehydes were formed to a higher extent. UV irradiation could induce the formation of bonds between the nuclei of aromatic residues, such as those of tyrosine and phenylalanine [33]. As a result, further cross-linking could proceed to a higher extent. Radiation cross-linking could improve the mechanical strength of reinforced polymers [34]. The results were consistent with the findings of Rhim et al. [13] and Gennadios et al. [35], who also observed improved mechanical properties and a decrease in EAB in various protein-based films after UV irradiation, which they attributed to the formation of cross-linked networks. Similar results were also observed in UV-treated films from sesame protein isolate [14] and whey protein concentrate [15].

When considering the effect of UV irradiation time, the longer time effectively induced cross-linking in films containing both LA and FeCl_2_ as indicated by the higher TS and EM. When comparing the UV irradiation methods (S-UV and F-UV), similar mechanical properties of films were obtained among the S-UV and F-UV films. Therefore, UV exposure time and intensity as well as the presence of LA and FeCl_2_ had a direct impact on the mechanical properties of the gelatin film.

#### 3.1.2. Water-Vapor Permeability (WVP)

**Figure 3:**
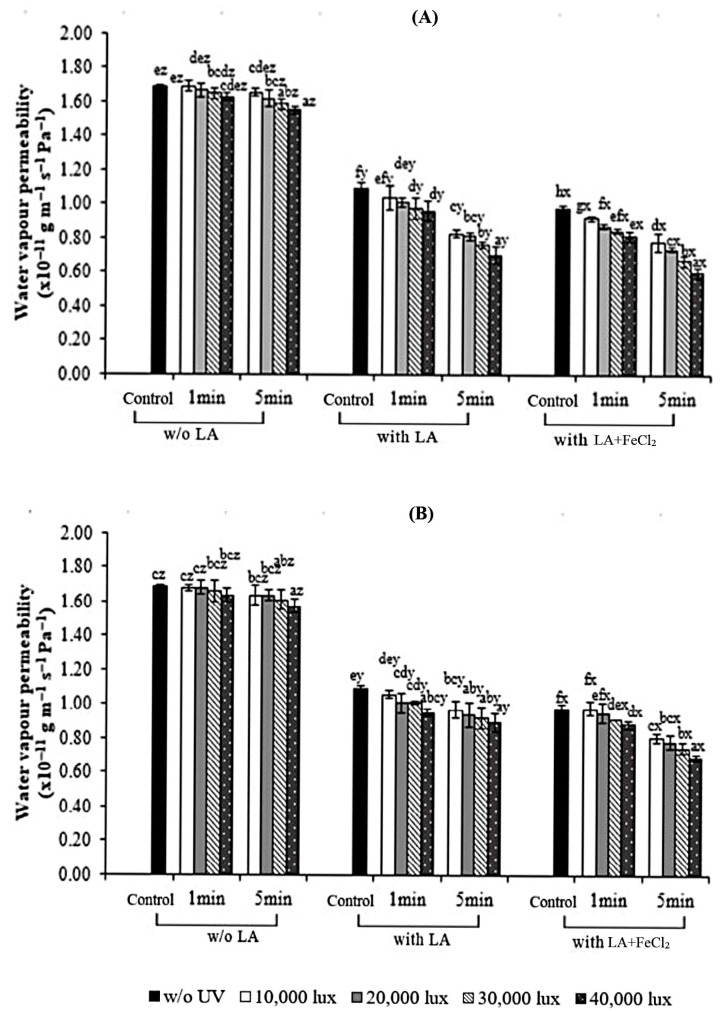
Effect of UV treatment by different irradiation methods on the water-vapor permeability (WVP) of gelatin films with and without 30% linoleic acid (LA) and 1.5% ferrous chloride (FeCl_2_). (**A**) S-UV: the film-forming solution was irradiated before casting; (**B**) F-UV: the pre-cast films were irradiated. The letters (a–h) and (x, y, z) on the bars indicate significant differences (*p* < 0.05).

The WVP of control gelatin, S-UV, and F-UV films without and with added LA and FeCl_2_ is shown in Figure 3. The WVP of the films was affected by LA, FeCl_2_, and UV radiation. For the gelatin film in which LA was incorporated, the WVP was decreased, irrespective of UV treatment. This more likely resulted from the high hydrophobic character of LA, which distributed in the film [36]. Including lauric acid (LA) in the gelatin film matrix decreased the film’s water-vapor permeability (WVP) regardless of the oil-to-protein ratio. The addition of other hydrophobic substances, such as palm oil, essential oil, and fatty acids, led to a reduction in WVP because of their inherent water-repelling properties [5,37]. Moreover, our results were consistent with the general understanding that the incorporation of hydrophobic substances, such as palm oil, essential oil, and fatty acids, leads to a lower WVP in gelatin films, as documented by Tongnuanchan et al. [27] and Limpisophon et al. [30].

Decreases in the WVP of the gelatin film samples were observed when UV irradiation with an increasing intensity level and time was implemented (*p* < 0.05), regardless of LA and FeCl_2_ incorporation. With increasing UV intensity and exposure time, the WVP of the resulting films decreased continuously. Increasing the light intensity during polymerization accelerates the reaction kinetics, leading to a more complete polymerization. This results in a final product with a lower concentration of unreacted functional groups [38]. Irradiation-generated hydroxyl and superoxide anion radicals can alter the molecular properties of proteins, which may lead to the formation of covalent cross-linkages. This process modifies the protein films [39]. A similar phenomenon is thought to occur in films treated with ultraviolet (UV) light.

The addition of LA in combination with UV treatment could significantly decrease the WVP of gelatin films. This observation indicates that UV irradiation more likely induced the cross-linking of film gelatin containing LA, especially in the presence of FeCl_2_. The exposure of proteins to UV radiation in the presence of LA may cause structural changes with increased protein surface hydrophobicity along with the attachment of oxidized LA into the gelatin chain [40]. The enhanced hydrophobicity of our films was also supported by Uchida et al. [41], who confirmed increased hydrophobicity of a poly(ethylene terephthalate) (PET) film upon UV treatment. Sabato et al. [42] reported that cross-linked soy and whey protein-based films treated with gamma radiation showed a decreased WVP in the presence of carboxymethylcellulose (CMC).

A further decrease was noticeable when FeCl_2_ was added in conjunction with LA (*p* < 0.05). The incorporation of FeCl_2_ could increase the rate of oil oxidation, producing lipid alkyl radicals [43]. Oxidized linoleic acid (OLA), an electrophilic compound, might be attached to a specific domain in gelatin [9,10]. This could enhance the cross-linking of protein in the film matrix, in comparison with a film without FeCl_2_ added. The WVP of the film with higher cross-linking had a higher decrease in WVP. The gelatin film with strong interaction of protein molecules in the matrix more likely had compactness. Together with the increased hydrophobicity, this resulted in the lower migration of moisture through the film [19]. When comparing the effect of UV irradiation on the WVP of S-UV and F-UV films, there was no difference in WVP (*p* ≥ 0.05). It can be inferred that the addition of LA and FeCl_2_ to the gelatin-based film subjected to UV at high intensity for a long time had a profound effect on the WVP of the film. Therefore, the results confirmed that UV irradiation is a viable method for improving the water vapor-barrier properties of packaging films including those from gelatin containing LA.

#### 3.1.3. Appearance and Color of Films

**Figure 4:**
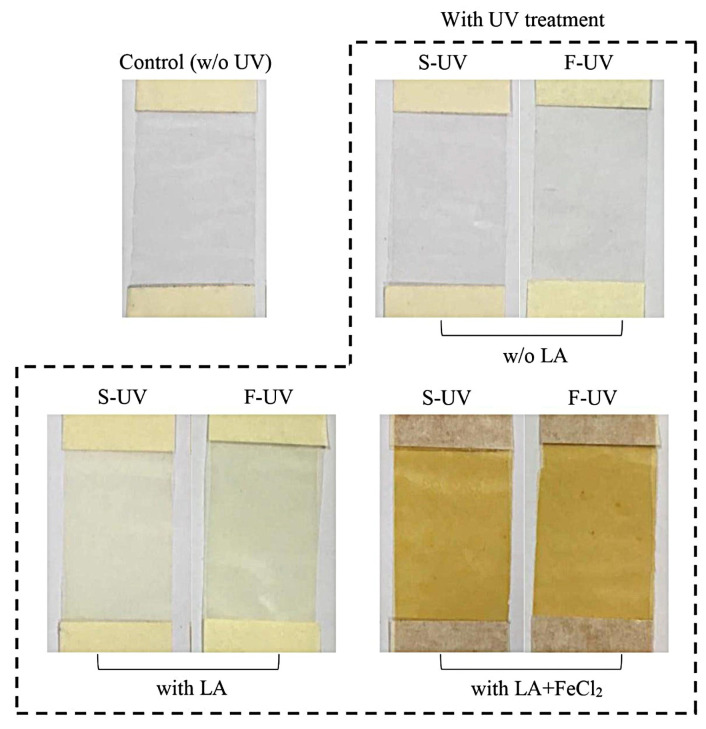
Photographs of selected films of fish skin gelatin with and without incorporation of 30% linoleic acid (LA) and 1.5% ferrous chloride (FeCl_2_) and irradiated with UV by different methods (S-UV and F-UV). S-UV: film-forming solution was irradiated prior to film casting; F-UV: pre-cast films were irradiated with UV directly; Control: gelatin film without UV treatment as well as LA and FeCl_2_ addition.

The appearance of the films from fish gelatin without and with the incorporation of LA and/or FeCl_2_ as affected by UV treatment is presented in Figure 4. The control gelatin film was colorless and transparent. After films were supplemented with LA, the film became turbid with slight yellowness. When FeCl_2_ was incorporated in combination with LA, the yellowness of the film was markedly increased. This was owing to the yellow color of FeCl_2_ in nature. When comparing the S-UV and F-UV films, no differences were detected visually.

**Figure 5:**
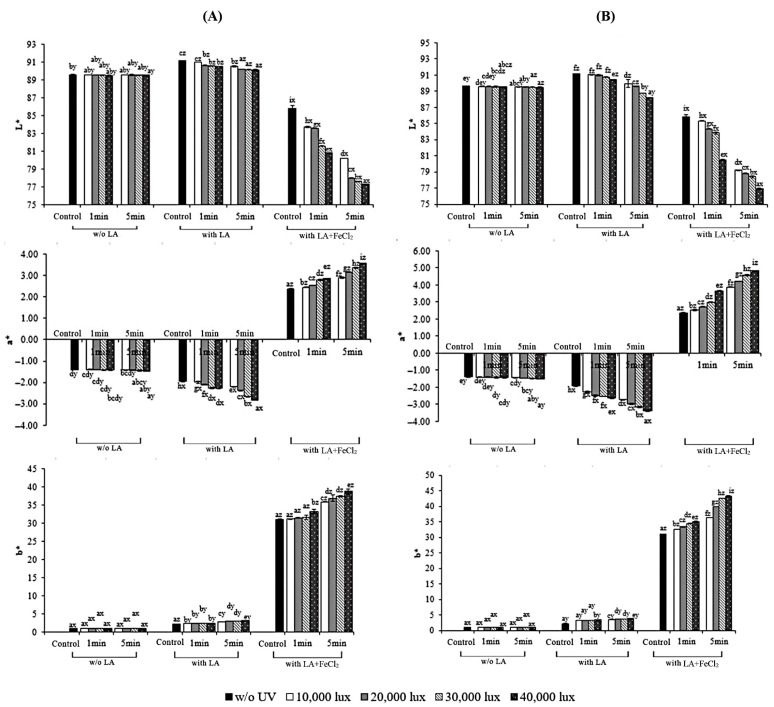
Effect of UV treatment by different irradiation methods on the color parameters of gelatin films with and without 30% linoleic acid (LA) and 1.5% ferrous chloride (FeCl_2_). (**A**) S-UV: the film-forming solution was irradiated before casting; (**B**) F-UV: the pre-cast films were irradiated. The letters (a–i) and (x, y, z) on the bars indicate significant differences (*p* < 0.05).

The color parameters of the films from fish skin gelatin with different treatments in comparison with the control are shown in Figure 5. Color is expressed as the L*-value (whiteness), a*-value (redness–greenness), and b*-value (blueness–yellowness). Generally, decreases in a*- but increases in L*- and b*-values were found when LA was incorporated (*p* < 0.05). This indicated that LA, having a yellow color, affected the color of the gelatin films. Similar results were reported for gelatin films in which various oils and fatty acids were incorporated [7,8,19]. On the other hand, substantial increases in a*- and b*-values with coincidentally lower L*-values were obtained for gelatin films supplemented with LA and FeCl_2_ (*p* < 0.05), compared with those of films from other treatments. It was suggested that the color of the films was significantly caused by FeCl_2_.

When UV irradiation was applied for either the FFS (S-UV) or pre-casted film (F-UV), the color was affected differently. The results showed that the b*- and a*-values were increased drastically when the exposure time and intensity of UV used for treatment increased (*p* < 0.05). Conversely, the L*-value decreased with increasing UV intensity and exposure time (*p* < 0.05). This finding suggested that the UV irradiation of a gelatin film, regardless of the stage for irradiation, resulted in an alteration in the color of all the film samples, in which yellow or brown color was detected. This color change is a visible indicator of the chemical reactions taking place. Oxidative products including aldehydes, ketones, hydrocarbons, esters, lactones, and alcohols in oxidized oil or fats as induced by UV light could interact with amino groups of gelatins, thus causing glycation or Maillard reactions [44]. The presence of these by-products from the cross-linking and protein glycation compounds were increased after UV treatment. Koh et al. [45] described that an increase in the yellowness of oxidized linoleic acid and linolenic acid was probably due to the thermally induced oxidation of fatty acids. However, the effect of UV irradiation on color was slightly higher when the treatment was applied to the dry film (F-UV). Masutani et al. [46] also documented a yellow color on the surface of gelatin films containing glucose subjected to UV irradiation. Therefore, both LA and FeCl_2_ together with UV irradiation were crucial factors affecting the appearance and color of the resulting gelatin films.

#### 3.1.4. Transparency Value

**Figure 6:**
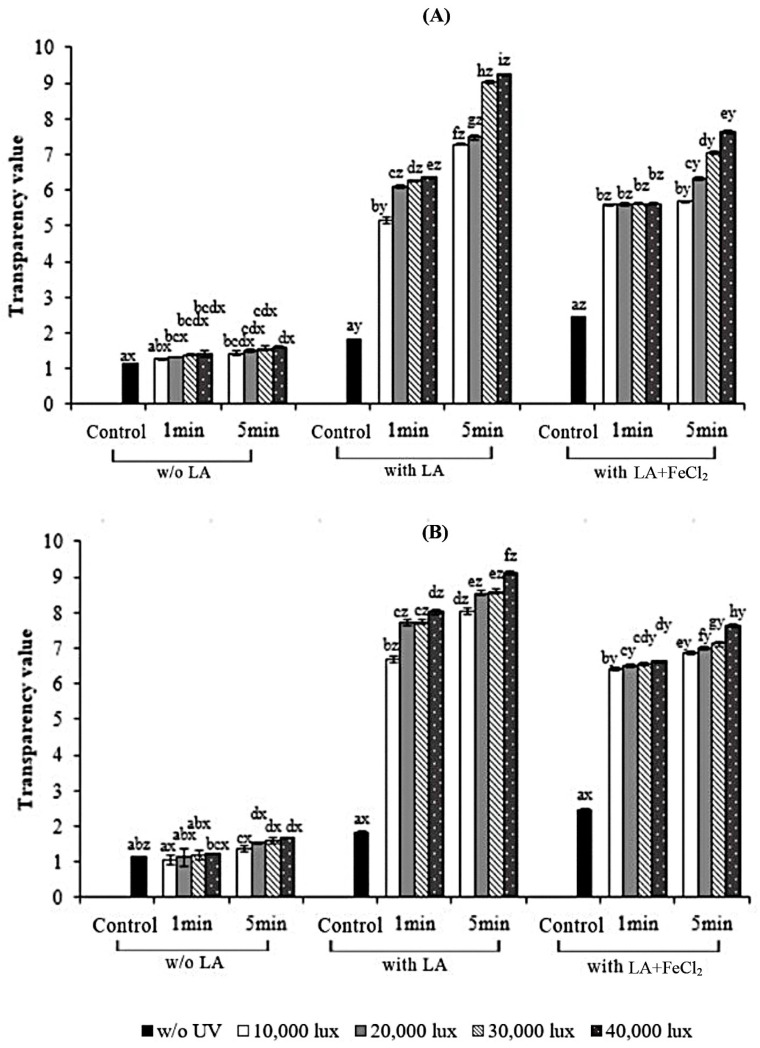
Effect of UV treatment by different irradiation methods on the transparency value of gelatin films with and without 30% linoleic acid (LA) and 1.5% ferrous chloride (FeCl_2_). (**A**) S-UV: the film-forming solution was irradiated before casting; (**B**) F-UV: the pre-cast films were irradiated. The letters (a–i) and (x, y, z) on the bars indicate significant differences (*p* < 0.05).

The transparency values of all the gelatin films as affected by the addition of LA and FeCl_2_ as well as exposure to UV radiation at different intensities and exposure times are depicted in Figure 6. The film sample supplemented with LA exhibited a higher transparency value than the control film (without LA) (*p* < 0.05). A higher transparency value was found for the gelatin film when both LA and FeCl_2_ were added (*p* < 0.05). This result indicated that the presence of fatty acids within the film matrix reduced the gelatin film’s transparency. The light-scattering effect caused by oil droplets significantly increased the film’s opacity. [19]. This result was in line with Pires et al. [47], who found that the incorporation of various essential oils (citronella, coriander, tarragon, and thyme oils) increased the transparency value of hake protein film. This confirms that the presence of lipid droplets causes a light-scattering effect, leading to reduced transparency.

The transparency value of the gelatin film increased and reached its highest value when irradiation was performed at 40,000 lux for 5 min, irrespective of the irradiation method (*p* < 0.05). The film’s transparency is influenced by its internal structure, as well as the amount and size of lipid aggregates that disperse within it during drying [48]. Similarly, it is observed that S-UV films had higher transparency values than F-UV films. It was presumed that UV could penetrate into the FFS easily, thus inducing the formation of aldehydes, and generated more free radicals than in the dry film. These free radicals, in turn, formed the additional cross-links as evidenced by the higher transparency value (corresponding to a less transparent film). A lower transparency value was found when no UV irradiation was implemented, similar to that reported by Kristo et al. [40].

### 3.2. Characteristics of Fish Skin Gelatin Films with Incorporation of Linoleic Acid (LA) and Ferrous Chlorides (FeCl_2_) Subjected to UV Irradiation Under the Selected Conditions

Some characteristics were evaluated for the S-UV and F-UV films containing and not containing 30% LA and 30% LA + 1.5% FeCl_2_, which were irradiated at a selected UV-irradiation intensity and exposure time of 40,000 lux and 5 min, respectively. The control gelatin film without UV irradiation as well as LA and FeCl_2_ addition was also characterized for comparison. The obtained results are as follows:

#### 3.2.1. Film Microstructure

**Figure 7:**
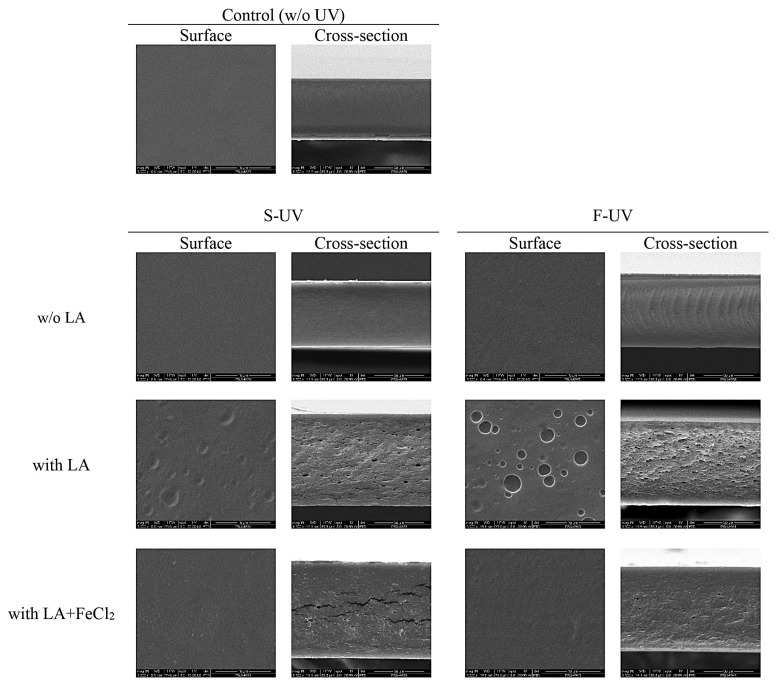
SEM micrographs of upper surfaces (5000×) and cross-sections (1500×) of selected films of fish gelatin with and without the incorporation of 30% linoleic acid (LA) and 1.5% ferrous chloride (FeCl_2_) and irradiated with UV (at 40,000 lux for 5 min) by different methods (S-UV and F-UV). S-UV: film-forming solution was irradiated prior to film casting; F-UV: pre-cast films were irradiated with UV directly; Control: gelatin film without UV treatment as well as LA and FeCl_2_ addition.

The morphologies of the surfaces and cross-sections of selected gelatin films with and without UV treatment are illustrated in Figure 7. Film samples treated with UV light, but without the addition of LA and FeCl_2_, had the smoothest and most uniform microstructure on both the surface and in cross-section compared to all the other UV-treated films. These films generally showed similar microstructures to the control film without UV treatment. However, adding LA to the film without FeCl_2_ resulted in the roughest surface. The film’s surface and cross-section also showed a high concentration of tiny droplets and pinholes. This result indicated that roughness was more likely due to the aggregation of lipid droplets.

The film containing FeCl_2_ as an oxidizing agent in the film sample and supplemented with LA exhibited a smoother surface without any voids and a dense structure on cross-section, compared with the film in which only LA was incorporated, followed by UV treatment. Overall, it had a similar surface microstructure to the control film (without LA and FeCl_2_ added). This result suggested that UV irradiation at high intensity and for sufficient time could induce interaction and cross-linking, especially when FeCl_2_ was introduced. As a result, the film matrix could be stabilized by a combination reaction including photo cross-linking, or intercross-linking may happen between the neighboring polymeric molecules [49]. The difference in structure between the UV-treated films without and with FeCl_2_ as an oxidizing agent could result from oxidatively induced interactions and cross-linking possibly occurring in the film matrix. Higher cross-linking between lipid oxidation products or generated radicals and gelatin molecules were found in the film prepared using FeCl_2_. The S-UV film containing LA + FeCl_2_ (S-UV3) had a more homogenous appearance of the surface and denser structure than the F-UV3 film. Thus, the microstructure of the film matrix was likely affected by molecular interactions in the gelatin films as governed by the different components used and UV irradiation.

#### 3.2.2. ATR-FTIR Spectroscopy

**Figure 8:**
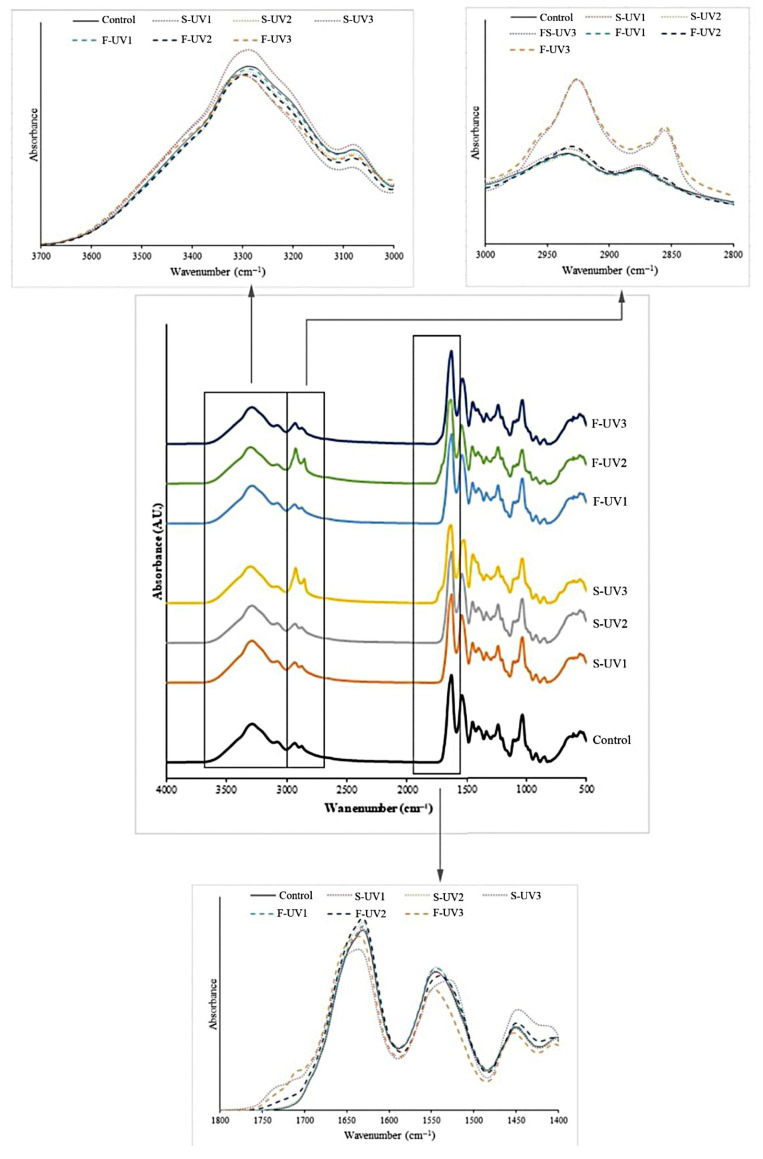
ATR-FTIR spectra of selected films of fish skin gelatin with and without incorporation of 30% linoleic acid (LA) and 1.5% ferrous chloride (FeCl_2_) and irradiated with UV (at 40,000 lux for 5 min) by different methods (S-UV and F-UV). S-UV: film-forming solution was irradiated prior to film casting; F-UV: pre-cast films were irradiated with UV directly; Control: gelatin film without UV treatment as well as LA and FeCl_2_ addition. The numbers 1, 2, and 3 following S-UV or F-UV refer to without LA and FeCl_2_, with 30% LA, and with 30% LA + 1.5% FeCl_2_, respectively.

Figure 8 illustrates the ATR-FTIR spectra of selected UV-irradiated gelatin films and the control gelatin film without UV irradiation and LA + FeCl_2_ addition. An FTIR measurement was performed to identify the chemical groups as well as possible chemical interactions and bonds responsible for the structural stabilization of the gelatin film matrix. The typical spectra characteristic of protein-based materials was generally observed in all the film samples. The absorbance bands of the fish gelatin film contained a series of the major bands at ~3280 cm^−1^ (amide-A, illustrating NH-stretching, coupled with hydrogen bonding), ~3079 cm^−1^ (representing stretching of CH in =CH or aromatic moiety and NH^3+^), ~2935 and 2867 cm^−1^ (amide-B, corresponding to asymmetric and symmetric stretching of aliphatic CH, respectively), ~1634 cm^−1^ (amide-I, representing the C=O stretching/hydrogen bonding coupled with COO-), ~1523 cm^−1^ (amide-II, illustrating the NH bending coupled with CN stretching), and ~1234 cm^−1^ (amide-III, representing the CN and NH in plane of amide bound or CH_2_ of glycine) [50]. The typical bands found in all the gelatin films aligned with those reported by Tongnuanchan et al. [19,27]. It could be noticed from the spectra that the amide-A band (~3290 cm^−1^) of UV-treated films generally showed a slight shift to lower wavenumbers (~3288–3285 cm^−1^) with a concomitant decrease in peak intensity as compared to the control film (untreated film). For the amide-B band (at ~2935 and ~2867 cm^−1^), a higher peak amplitude was observed in UV-irradiated films containing LA (S-UV2, F-UV2) and LA + FeCl_2_ (S-UV3, F-UV3), which was more intense in the later film samples, in comparison with the control film. The wavenumbers at ~2935 and ~2867 cm^−1^ were generally related to alkyl groups and were also reported to correlate with the increased hydrophobicity of molecules [51]. Similar results were observed by Wu et al. [52], who pointed out that the presence of LA under UV irradiation could enhance the presence of hydrophobic groups in the gelatin film. In addition, irrespective of the irradiation method, the incorporation of FeCl_2_ into films containing LA that were treated with UV could further enhance the interaction and cross-linking between gelatin chains, possibly induced by UV–oxidative reactions. This could consequently decrease hydrophilic moieties and hence increase the hydrophobicity of the films. These results align with the WVP decrease observed in S-UV and F-UV films that incorporated LA + FeCl_2_ (Figure 3).

In the amide-I and -II regions, a shift to lower wavenumbers for these peaks could be noticed for films with UV irradiation, regardless of LA and LA + FeCl_2_ addition. A remarkable shift of amide-II to lower wavenumbers (1544 to 1528 cm^−1^) was observed for the S-UV-treated gelatin film with the incorporation of LA and FeCl_2_ (S-UV3). The shift in the amide-I and -II peaks of gelatin could relate to the conformational change involving the helix–coil structure as well as the formation of hydrogen bonding and other kinds of bonding and interaction [52]. Furthermore, the intensity of the amide-II peak decreased when UV irradiation was implemented, irrespective of the S-UV and F-UV methods, especially in the films with LA and FeCl_2_ addition. The decrease in the intensity of amide-I, amide-II, and amide-A could suggest that UV treatment affected the interaction of amino groups as well as amide carbonyl groups and induced the cross-linking of proteins [15]. The shifts in amide-I and amide-II peaks and the decrease in their intensity, which was attributed to UV-induced cross-linking and interaction with lipid oxidation products, were consistent with the findings of other studies on protein modification. For instance, a decrease in the intensity of amide-I and amide-II peaks after UV treatment was also reported by Wang et al. [53] for fish gelatin films, supporting our conclusion that UV irradiation promotes protein cross-linking.

The increase in cross-linking might be due to the generated peroxide radicals and other reactive species [54]. Generally, LA is prone to oxidation due to the presence of double bonds. Similarly, the double bonds and aromatic rings in amino acids like tyrosine and phenylalanine can absorb UV radiation. This absorption can generate free radicals, leading to the formation of intermolecular covalent bonds [13,14]. Moreover, it could be noticed that the F-UV film containing LA + FeCl_2_ (F-UV3) showed not only a remarkable reduction in the intensity of amide-I and II but also a significant increase in the intensity of the peak in the wavenumber range of 1455–1400 cm^−1^, characteristic of the C-N bond, when compared to other samples. This could be indicative for additional covalent cross-linking possibly mediated via bonding between the carbon atoms of reactive species generated from lipid oxidation and the NH group of gelatins. It should be noted that the incorporation of FeCl_2_, a pro-oxidant, in the film was accompanied by an increase in cross-linking between gelatin and LA molecules, particularly when the film was exposed to UV irradiation. This was likely due to covalent bond formation between the ɤ-carbonyl group in LA residues and the Ɛ-amino group of a lysine residue [9]. This obtained result strongly supported the highest increase in strength and decrease in WVP found in the S-UV film containing LA + FeCl_2_ compared to other films (Figure 2 and Figure 3).

Furthermore, the existence of a peak at around 1715–1765 cm^−1^ was observed in LA-supplemented films with UV irradiation. This peak was more likely caused by the stretching vibration of the C=O in aldehydes or carbonyl-containing species [55] generated from UV-induced oxidation. This was more pronounced when FeCl_2_ was incorporated. These reactive species could undergo reactions with gelatin, resulting in the cross-linking of gelatin. In general, the FTIR spectra of films prepared by different UV irradiation methods (S-UV and F-UV) were very similar. However, the changes, such as the shift and decrease in the intensity of amide-I and amide-II as well as the increase in the intensity of the peak around 1455–1400 cm^−1^, were more pronounced and clearly noticed in S-UV films, especially with FeCl_2_ addition. In the solution state, the molecules had higher mobility and the UV radiation applied might have been able to penetrate into the film-forming solution easily. This could have induced the formation of aldehydes and generated more free-radicals than in the dry film. Correspondingly, the cross-linking of gelatin induced by oxidation and UV irradiation could take place to a higher extent in the film-forming solution as compared to the dry film of gelatin. Therefore, the incorporation of LA and FeCl_2_ followed by UV irradiation could provide hydrophobic groups and induce protein cross-linking in the resulting films. The more hydrophobic substance resulted in a decrease in the WVP of the film. Similarly, the higher interaction between proteins in the fish gelatin film matrix might be one of the major causes of the increases in TS and EM.

#### 3.2.3. TGA

**Figure 9:**
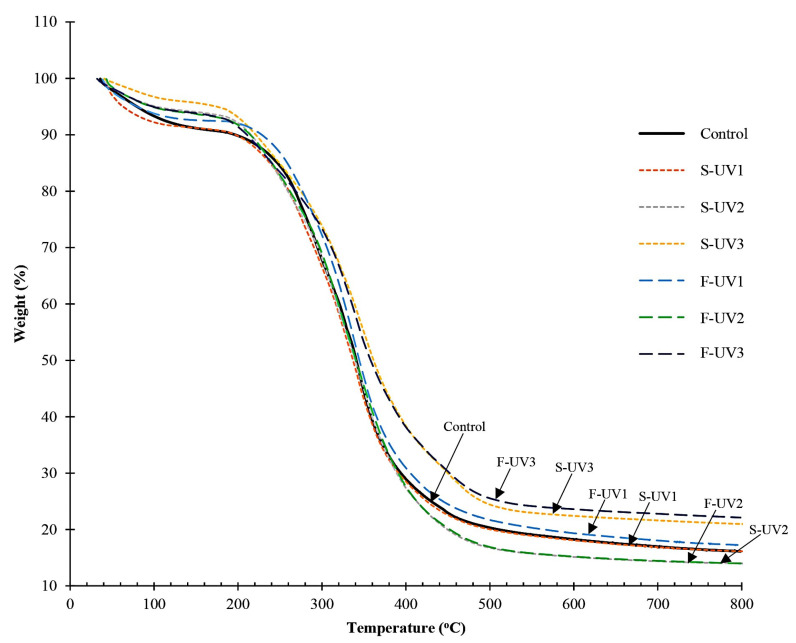
TGA curves of selected films of fish skin gelatin with and without incorporation of 30% linoleic acid (LA) and 1.5% ferrous chloride (FeCl_2_) and irradiated with UV (at 40,000 lux for 5 min) by different methods (S-UV and F-UV). S-UV: film-forming solution was irradiated prior to film casting; F-UV: pre-cast films were irradiated with UV directly; Control: gelatin film without UV treatment as well as LA and FeCl_2_ addition. The numbers 1, 2, and 3 following S-UV or F-UV refer to without LA and FeCl_2_, with 30% LA, and with 30% LA + 1.5% FeCl_2_, respectively.

Thermogravimetric analysis was employed to determine the thermal stability of the material. The thermal stability of any polymeric material is largely determined by the strength of the covalent bonds between polymer molecules. TGA thermograms of different selected films are shown in Figure 9. The temperature of degradation, weight loss, and residue mass are provided in Table 1. The TGA thermogram of all the samples generally exhibited three zones of weight loss. Those changes included the first step, in a temperature range of 50.72–58.47 °C (T_d1_) with a weight loss of 4.07–9.20% (∆w_1_); the second step, in a temperature range of 193.25–246.38 °C (T_d2_) with a weight loss of 12.35–30.44% (∆w_2_); and the third step, in a temperature range of 316.10–382.80 °C (T_d3_) with a weight loss of 42.15–62.32% (∆w_3_). The first stage of weight reduction observed in all the films was mostly linked to the loss of moisture and other low-molecular-weight volatile compounds within their structure. This initial weight loss ascribed to the evaporation of free water was typically observed in various bio-based films including from gelatin [27,28]. It was noticed that films supplemented with LA and subjected to UV irradiation had lower weight loss than the control film (w/o UV), caused by the presence of a hydrophobic substance in the film [27], especially when FeCl_2_ was present.

The second weight loss periods of all the films were detected in a temperature range of 193.25–246.38 °C (T_d2_), with a weight loss of 12.35–30.44% (∆w_2_). This degradation temperature could result from the decomposition of the gelatin film matrix and the constituents, more likely the small molecule fractions.

The thermal stability of the gelatin film was compromised by the loss of its glycerol plasticizer and the degradation of a low-molecular-weight protein [56]. Similarly, Tongnuanchan et al. [18] found that adding citrus essential oil to fish skin gelatin films led to reduced degradation temperatures and increased weight loss compared to pure gelatin films. For UV-treated samples, a lower T_d2_ degradation temperature was found, compared to the control film (w/o UV). The UV treatment might have caused the oxidative degradation of gelatin chains to the same degree as well as generating reactive lipid oxidation products with small molecules, especially when LA and FeCl_2_ were incorporated. These low-molecular-weight fractions produced in the UV-treated films could be thermally degraded at a lower T_d2_ temperature compared to those of the control film without UV treatment. The temperature range for the third step of thermal degradation (T_d3_) appeared at 316.10–382.80 °C with a weight loss (∆w_3_) of 42.15–62.32%, suggesting the depolymerization of a large molecule fraction of gelatin chains in the film network. A higher degradation temperature and weight loss in this T_d3_ range were obtained in UV-exposed films with LA incorporation, especially when FeCl_2_ was incorporated, indicating that the film structure was more thermo-stable than the other films. The formation of thermo-stable fractions was mostly correlated with the oxidation induced by UV irradiation. FeCl_2_ could increase the oxidation of LA, leading to enhanced cross-linking between LA and gelatin chains, possibly involving covalent bonds, in which a strong matrix was formed. The covalent cross-linked fractions with thermal resistance could be formed to a higher extent in the UV-treated film containing LA and FeCl_2_ as a pro-oxidant, resulting in the highest T_d3_ of this film. This was also in accordance with the highest residue mass (representing char content) observed in the UV-treated film containing LA and FeCl_2_, compared with the other films. The film containing both LA and FeCl_2_ had the highest lipid oxidation products or radicals, which could lead to the cross-linking of protein in the film matrix, as evidenced by the IR result (Figure 8). Thus, the use of UV treatment for a gelatin film containing both LA and FeCl_2_ had a marked impact on the thermal degradation behavior and stability of the resulting gelatin film.

#### 3.2.4. Protein Pattern

**Figure 10:**
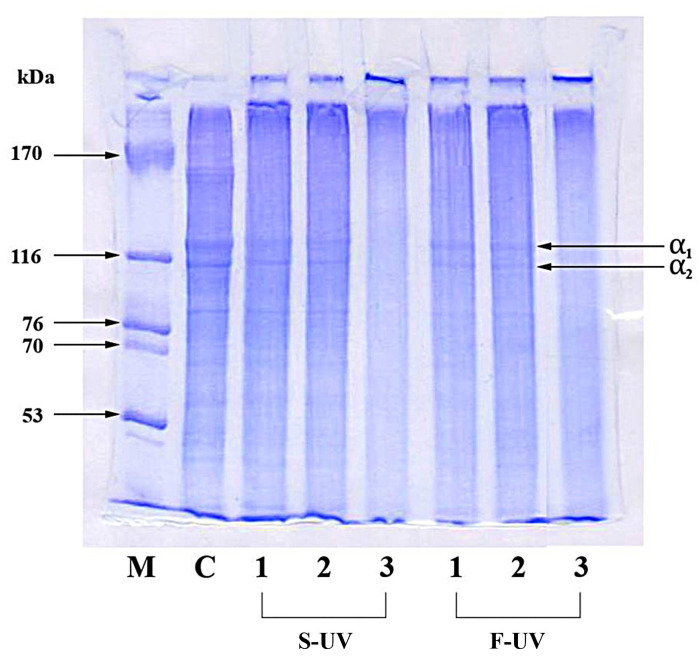
Electrophoretic profiles of selected films of fish gelatin with and without incorporation of 30% linoleic acid (LA) and 1.5% ferrous chloride (FeCl_2_) and irradiated with UV (at 40,000 lux for 5 min) by different methods (S-UV and F-UV). S-UV: film-forming solution was irradiated prior to film casting; F-UV: pre-cast films were irradiated with UV directly; Control: gelatin film without UV treatment as well as LA and FeCl_2_ addition. The numbers 1, 2, and 3 following S-UV or F-UV refer to without LA and FeCl_2_, with 30% LA, and with 30% LA + 1.5% FeCl_2_, respectively.

The SDS-PAGE protein patterns of different films (S-UV- and F-UV-treated films), with and without LA and LA + FeCl_2_, are shown in Figure 10. Generally, the primary components found in fish skin gelatin are the α_1_- and α_2_-chains [57]. For films prepared from the FFS subjected to UV light before casting (S-UV), the protein pattern of gelatin films without and with LA after UV irradiation (S-UV1 and S-UV2) was slightly different from that of the control film. The band intensities of the α_1_- and α_2_-chains were much lower, compared with those found for the control film. The data suggested that UV irradiation can induce cross-linking in the film matrix, leading to an augmentation in the molecular weight of the proteins in the resulting gelatin film. There was no marked difference in protein pattern between the film prepared from the FFS subjected to UV irradiation in the absence (S-UV1) and presence of LA (S-UV2). The results indicated that the addition of LA into the gelatin film-forming solution followed by UV treatment had no effect on the increase in the molecular weight of the gelatin in the film. However, drastic differences in protein patterns were observed between the film from the FFS containing LA (S-UV2) and film containing LA + FeCl_2_ (S-UV3) after UV treatment, by the disappearance of the bands of α_1_- and α_2_-chains. This phenomenon was owing to the enhanced cross-linking of gelatin in the film matrix via non-disulfide covalent bonds. FeCl_2_ was able to induce LA oxidation via UV irradiation. The cross-link formation in irradiated proteins significantly affected the structural organization of macromolecules [58].

In general, the stage or method of UV irradiation (S-UV and F-UV) had no profound impact on the protein pattern of the resulting films. There was no marked difference between films prepared from the FFS UV irradiated before casting (S-UV) and film directly subjected to UV irradiation (F-UV) when the same additive (LA or LA + FeCl_2_) was used. Therefore, LA in combination with FeCl_2_ could induce non-disulfide covalent bonds between α-chains of gelatin, causing the changes in the properties of the resulting gelatin films.

## 4. Conclusions

The incorporation of both LA and FeCl_2_ significantly improved the water-vapor barrier property, while mechanical performance was also enhanced through UV irradiation. This was mainly associated with the increased protein cross-linking from non-disulfide covalent bonds. However, the yellowness of the treated film was increased, whereas the film became less transparent. The stage of UV irradiation had no influence on the properties of the film; therefore, it could be implemented in either the film-forming solution or the pre-cast film. Based on the improved properties, the developed gelatin films show great potential for application as food packaging materials. These films are particularly suitable for packaging dry and high-fat foods, such as nuts, dried fruits, potato chips, crackers, and fatty powders, helping to extend their shelf life. The increased mechanical strength also makes the films more durable and resistant to handling during storage and transport. Considering the trade-off between properties, the incorporation of LA and FeCl_2_ into a fish gelatin film that is UV irradiated at an intensity of 40,000 lux for 5 min in the film-forming solution prior to casting is suggested to improve the water-vapor barrier property of the fish gelatin film for these specific food packaging applications.

## Figures and Tables

**Table 1:** Thermal degradation temperatures (T_d_, °C) and weight loss (Δ*w*, %) of selected films of fish skin gelatin with and without incorporation of 30% linoleic acid (LA) and 1.5% ferrous chloride (FeCl_2_) and irradiated with UV (at 40,000 lux for 5 min) by different methods (S-UV and F-UV).

Figure 2	LA (%)	FeCl_2_ (%)	∆_1_		∆_2_		∆_3_		Residue (%)
			T_d1_	∆*w*_1_	T_d2_	∆*w*_2_	T_d3_	∆*w*_3_	
Control	–	–	56.74	9.20	246.38	30.44	334.08	42.15	18.21
S-UV1	–	–	54.79	8.56	233.14	20.77	321.95	52.51	18.16
S-UV2	30	–	50.72	5.71	205.41	17.30	344.50	61.29	15.70
S-UV3	30	1.5	58.47	4.07	193.25	14.77	382.80	57.75	23.41
F-UV1	–	–	54.13	7.35	243.58	17.65	316.10	55.71	19.29
F-UV2	30	–	56.07	6.28	211.31	15.83	329.58	62.32	15.57
F-UV3	30	1.5	57.03	5.90	194.45	12.35	372.27	57.88	23.87

∆_1_, ∆_2_, and ∆_3_ represent the first, second, and third periods of weight loss, respectively, of the film sample during the heating scan (50–800 °C). Control: control gelatin film without UV irradiation as well as LA and FeCl_2_ addition. S-UV: film-forming solution was exposed to UV irradiation prior to casting; F-UV: pre-cast film was exposed to UV irradiation. Numbers 1, 2, and 3 following S-UV or F-UV denote samples without LA and FeCl_2_, with LA, and with LA + FeCl_2_, respectively.

## Data Availability

The original contributions presented in this study are included in the article. Further inquiries can be directed to the corresponding author.
